# Newly Exerted T Cell Pressures on Mutated Epitopes following Transmission Help Maintain Consensus HIV-1 Sequences

**DOI:** 10.1371/journal.pone.0120787

**Published:** 2015-04-28

**Authors:** Emily M. Eriksson, Teri Liegler, Chris E. Keh, Annika C. Karlsson, Sara J. Holditch, Christopher D. Pilcher, Lisa Loeb, Douglas F. Nixon, Frederick M. Hecht

**Affiliations:** 1 Division of Experimental Medicine, Department of Medicine, University of California, San Francisco Positive Health Program, San Francisco General Hospital, San Francisco, California; 2 Department of Medicine, University of California, San Francisco Positive Health Program, San Francisco General Hospital, San Francisco, California; 3 Department of Laboratory Medicine, Division of Clinical Microbiology, Karolinska Institutet, Stockholm, Sweden; Institute of Infection and Global Health, UNITED KINGDOM

## Abstract

CD8^+^ T cells are important for HIV-1 virus control, but are also a major contributing factor that drives HIV-1 virus sequence evolution. Although HIV-1 cytotoxic T cell (CTL) escape mutations are a common aspect during HIV-1 infection, less is known about the importance of T cell pressure in reversing HIV-1 virus back to a consensus sequences. In this study we aimed to assess the frequency with which reversion of transmitted mutations in T cell epitopes were associated with T cell responses to the mutation. This study included 14 HIV-1 transmission pairs consisting of a *‘source’* (virus-donor) and a *‘recipient’* (newly infected individual). Non-consensus B sequence amino acids (mutations) in T cell epitopes in HIV-1 *gag* regions p17, p24, p2 and p7 were identified in each pair and transmission of mutations to the recipient was verified with population viral sequencing. Longitudinal analyses of the recipient’s viral sequence were used to identify whether reversion of mutations back to the consensus B sequence occurred. Autologous 12-mer peptides overlapping by 11 were synthesized, representing the sequence region surrounding each reversion and longitudinal analysis of T cell responses to source-derived mutated and reverted epitopes were assessed. We demonstrated that mutations in the source were frequently transmitted to the new host and on an average 17 percent of mutated epitopes reverted to consensus sequence in the recipient. T cell responses to these mutated epitopes were detected in 7 of the 14 recipients in whom reversion occurred. Overall, these findings indicate that transmitted non-consensus B epitopes are frequently immunogenic in HLA-mismatched recipients and new T cell pressures to T cell escape mutations following transmission play a significant role in maintaining consensus HIV-1 sequences.

## Introduction

The genetic composition of the HIV-1 virus is continuously evolving. The relatively high level of errors introduced by the HIV-1 reverse transcriptase and recombination both contribute to HIV-1 polymorphism. These mutations provide a means to evade CTL pressure and T cell recognition in the host [1–3]. Although most transmitted mutations persist in the new host early after transmission, transmission to a new host with different HLA types often results in a situation in which host CTL pressures that had previously selected for an escape mutation are no longer present. Once host CTL selection pressure favoring a particular mutation is removed, some CTL escape mutations persist, while others revert over time [4–7]. The viral fitness cost has been shown to drive the reversion of some transmitted CTL escape mutations [4, 8]. However, host responses directed to variant epitopes have also been observed [9, 10], where CTL escape mutations from an earlier host may also serve as epitopes that induce immune responses in the context of different HLA types, driving reversion to consensus sequences. We focused our study on HIV-1 gag sequence as this protein is rich in CTL epitopes and is one of the most conserved viral proteins. This makes it a suitable region in which to assess the relative contribution of newly induced immune responses in driving loss of transmitted CTL escape mutations, an area that remains controversial. To better understand the role of immune pressure in reversions, this study examined T cell responses to transmitted mutations in a new host. Our data provide further support to the concept that persistence of many mutated epitopes following transmission to a new host is limited due to back selection of the virus to consensus sequence, which is in part due to T cell immune pressures on variant epitopes.

## Methods

### Study subjects

We studied 14 HIV-1 transmission pairs recruited by the University of California at San Francisco (UCSF) Options Project. The Options Project enrolls subjects with recent HIV-1 infection (≤ 6 months), based on recent negative HIV-1 antibody tests, recent illness compatible with acute retroviral syndrome and serologic testing consistent with recent HIV-1 seroconversion, or HIV-1 RNA level > 3,000 with a negative HIV-1 antibody test [11]. The Options Project performed detailed interviewing to identify possible transmission partnerships in which a source might have transmitted HIV-1 to the newly infected case (recipient). We used phylogenetic analysis of *pol* consensus sequences (TRUGENE, Siemens Healthcare Diagnostics, Inc. Tarrytown, NY) to determine genetic relatedness of source and recipient viruses. Transmission pairs were defined if viruses clustered on the same tree branch with a bootstrap value of ≥700 (70%) and a difference in branch length representing less than approximately 6% of genetic difference [12]. Plasma and cryopreserved peripheral blood mononuclear cells (PBMCs) from 14 HIV-1 transmission pairs with phylogenetic evidence supporting transmission were studied. The individuals who transmitted the HIV-1 virus to their partners were designated ‘sources’ and the newly infected individuals were referred to as ‘recipients’. All sources were not on antiretroviral therapy (ART) at the time of sample collection and all recipient samples were collected while the individuals were treatment naïve. Population sequencing of *gag* regions p17, p24, p2 and p7 was performed on plasma virus from both sources and recipients. Additional population sequence genotyping was performed in the newly infected individuals at the latest time point available prior to commencement of ART. This follow-up time point was a mean of 185.2 weeks after the estimated date of transmission (range 34 to 339 weeks). PBMC from both sources and recipients used for immunological assays were from time points that coincided with the population sequencing. Longitudinal responses in the recipients were assessed using additional PBMC from time points at 12 month intervals until ART commencement. This study was approved by the UCSF Committee on Human Research (CHR#10–00301) and all subjects gave informed written consent to participate in this study, in accordance with the Declaration of Helsinki.

### HIV-1 *gag* amplification and sequencing from viral RNA

Viral RNA from 1.0 mL ACD or EDTA blood plasma was isolated using QIAamp Viral RNA kits (Qiagen, Valencia CA). HIV-1 *gag* regions encoding p17, p24, p2, p7 (HXB2 nt 737 through 2096) were sequenced using a nested, two-step RT-PCR procedure. HIV-1 cDNA was generated from 8 μL viral RNA using Thermoscript RT combined with Superscript II (Invitrogen Life Technologies, San Diego, CA) and primer RA01 (5’-CTGCTCCTGTATCTAATAGAGCTTC-3’; positions 2313–2337). A 1.6 kb amplicon was generated from 5.0 μL cDNA using the FastStart PCR kit (Applied Biosystems, Foster City CA), primers 737F (5’-GCGACTGGTGAGTACGCC-3’; positions 737–754) and RA01 under the following conditions: 94°C x 2’, 35(94°C x 30”, 56°C x 30”, 68°C x 2‘), 68°C x 4‘, 10°C hold. 1 μL of the PCR product was used for the nested PCR using primer pairs 737F and JA155mod (5’-CTGATAATGCTGAAAACATGGGTA-3’; positions 1295–1318), 1232F (5’- ACCTAGAACTTTAAATGCATGGG-3’; positions 1233–1255) and 1754R (5’- CAACAAGGTTTCTGTCATCC-3’; positions 1736–1755), 1503F (5’-GGAAGTGACATAGCAGGAA-3’; positions 1486–1504) and 2095R (5’-TTCCCTAAAAAATTAGCCTG-3’; positions 2077–2096) using the following conditions: 94°C x 2’, 30(94°C x 30”, 56°C x 30”, 68°C x 30’), 68°C x 4‘, 10°C hold. PCR products were treated with ExoSAP-IT (GE Healthcare Life Sciences) using manufacturer’s conditions and quantified by PicoGreen (Invitrogen/Life Technologies). The nested PCR primers also served as the sequencing primers using BigDye v3 cycle sequencing chemistry (Applied Biosystems/Life Technologies). Amplicon sequences for each sample were then determined using a 3130xl capillary array sequencer (Applied Biosystems/Life Technologies) following the manufacturer’s recommended conditions. Forward and reverse sequences were analyzed and edited using Sequencher (v. 4.6, Genecodes, Ann Arbor MI).

### Peptides

Autologous 12-mer peptides overlapping by 11 amino acids were synthesized (New England Peptide, Gardner, MA). Overlapping peptides spanned a sequence region of a total of 17 amino acids representing 8 amino acids upstream and 8 amino acids downstream of each identified reversion position in each recipient.

### ELISPOT assay

Peptide-specific T cell responses were assessed by IFN-γ ELISPOT assays as described previously [13]. PBMCs were stimulated with either autologous 12-mer HIV-1 Gag peptides (5 μg/ml) or phytohaemagglutinin (PHA) (1 μg/ml, Sigma, St Louis, MO). Spot quantification was achieved with an AID automated ELISpot reader (Cell Technology International, Columbia, MD). For HIV-1-specific responses, average spot counts for duplicate wells were calculated and background from wells with cells in media only was subtracted. Wells containing above 50 spot forming units (SFU) following subtraction of two times background were considered a positive response.

## Results

### Non-consensus viral sequences are transmitted at high frequencies to a new host

We first assessed whether T cell epitopes in which sequence variants differing from consensus were transmitted from the source to the recipient, denoted hereafter as ‘mutations.’ Many of these represent mutations that result in escape from CTL pressures. As some CTL escape mutations may impair viral fitness and the potential for transmission, one mechanism for maintaining consensus sequences may be selection pressures that favor initiation of a new infection by virus with consensus sequence. We sequenced HIV-1 *gag* regions encoding p17, p24, p2, p7 from both sources and recipients. Sequences were derived from each transmission pair on an average of 11 weeks (range 3.4 to 21.5 weeks) following estimated transmission. To establish the occurrence of non-consensus B sequence amino acids in *gag* T cell epitopes, sequences from each HIV-1 infected individual were compared to consensus B sequence from the Los Alamos HIV Sequence Database (hiv-web.lanl.gov). The overall frequency of mutations observed in the source, was an average of 21.9 ± 8.1.6 mutations per sequence. An average of 4.9 (range 0 to 12) new mutations that were not present in the source were observed in the recipients. To determine the transmission frequency of mutations, sequences from the source and the corresponding recipient were compared within each transmission pair and a transmitted mutation was identified as an amino acid mutation present in both the source and the recipient. The mean transmission frequency of mutations was 85.6% ± 11.2.

### Identification of reversions and determination of reversion rates

To establish the rate of reversions back to consensus B amino acids in the recipient, we performed population HIV-1 *gag* sequence genotyping in the recipient at the latest time point available before commencement of ART. This follow-up time point was a mean of 185.2 weeks after the estimated date of transmission (range: 34 to 339 weeks). Amino acids that had previously been identified in the source as mutations at the time point close to estimated transmission were examined for presence in the recipient. Amino acids in the recipient that were the same as consensus B, but differed in the source were defined as ‘reversions’. On average there were 4.3 ± 1.8 reversions in the recipient group at the last time point. Eighteen percent of the reversions were polymorphic sites suggestive of a mixed population of viruses containing either the reverted amino acid or the mutated amino acid. In 5 recipients a reverted amino acid was identified at the early time point but had mutated back to the original mutation present in the source partner at the later time point.

### Reversions abrogate T cell recognition

**Fig 1:**
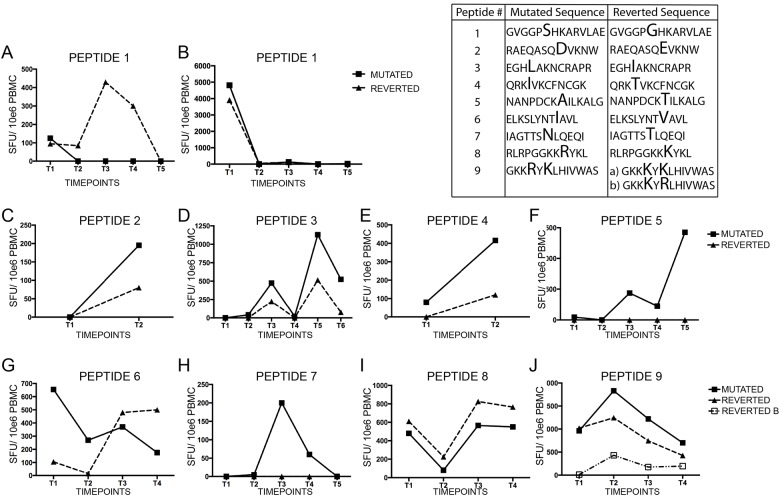
Summary of the magnitude of T cell responses. T cell responses were measured by IFN-γ production following stimulation with autologous 12-mer peptides. (A-J) Longitudinal analysis of T cell responses to both mutated and reverted peptides in separate recipients.

An escape mutation in a T cell epitope in one host may represent an immunogenic sequence in a new host with a different HLA type, resulting in immunologic pressures that select for viral variants containing consensus sequences. To assess the role of T cell responses in the new host in driving loss of these potential T cell escape mutations, we assessed responses in the recipients from initial recipient samples, which were collected at a median of 16 weeks post-transmission (range 3.4 to 35 weeks) and annually thereafter until commencement of ART. T cell responses to autologous peptides containing the mutated amino acid were observed at one or more time-points in 7 of the 14 recipients (Table 1), representing 9 sequence regions (Table 2). Due to limited cell availability, source responses were assessed at a single time point as close as possible to the time of estimated transmission. Only one of the peptides recognized in recipients was also recognized in a corresponding source (data not shown). For the nine sequence regions in which we detected T cell responses in the recipient to a transmitted mutated epitope, we compared the magnitude of T cell responses of the mutated epitope to the reverted consensus sequence epitope. Using an ELISPOT assay, we found that the number of IFN-γ producing cells responding to the consensus sequence epitope was decreased by 50% or more in 5 individuals (Fig 1C–1H). Longitudinal analysis in these recipients demonstrated that the responses to the peptides containing reverted amino acids were consistently lower than to the mutated version over time (Fig 1C–1F and 1H). Responses to two of the reverted peptides were completely absent whereas responses to the corresponding mutated peptide was maintained over several time points (Fig 1F and 1H). This suggests that reversion back to consensus B amino acids efficiently abrogates T cell recognition to these epitopes in the new hosts. In contrast, responses to 2 reverted peptide sequences (Fig 1A,1B and 1I; Peptide 1 and 8) were either equal or higher than responses to the corresponding mutated peptides, suggesting that reversion in these cases did not result in loss of T cell recognition. Additionally, the peptide containing the HLA-A2 restricted epitope SLYNTVAVL (Peptide 6) and the analogous mutated amino acid version SLYNTIAVL both induced T cell IFN-γ production. However responses to the mutated peptide dominated at the early time points whereas responses to the reverted peptides increased over time (Fig 1G). We also observed in one recipient that the response was diminished by reversion, but IFN-γ production was further decreased following introduction of a second mutation (Fig 1J). Overall, these data suggest that back selection to consensus B sequence diminishes T cell recognition in a new host for certain epitopes.

**Table 1:** Subject HLA information of responders.

Subject HLA information			
Source (S) Recipient (R)	HLA-A	HLA-B	Peptide Sequence responses
S1	24/33	39/14	
R1	03/03	07/40	Peptide 1
S2	01/24	35/57	
R2	02/03	07/27	Peptide 1,5,7
S3	01/01	08/57	
R3	11/31	44/56	Peptide 2
S4	0201*/25	39/51	
R4	0201*/31	44/44	Peptide 3
S5	02/24	27/44	
R5	11/01	08/52	Peptide 4
S6**	0201*/0202*	1503*/53	
R6	11/24	13/48	Peptide 9
R7	0201*/0203*	1501*/35	Peptide 6, 8

* Four digit HLA typing where HLA alleles matched between source and recipient

** Source transmitted HIV-1 virus to two different recipients

**Table 2:** HLA restriction of peptide sequences which are immunogenic in the recipients.

Peptide #	SEQUENCES		Protein region	Associated HLA restriction
1	REVERTED	GVGGP**G**HKARVLAE	p24	A2, A11, B07, B8, B35, B42, B81
	MUTATED	----------**S**----------------		
2	REVERTED	RAEQASQ**E**VKNW	p24	A11, B44, B45, B51, B57, Cw4, Cw8
	MUTATED	--------------**D**---------		
3	REVERTED	EGH**I**AKNCRAPR	p7	A3, A30, A31
	MUTATED	------**L**---------------		
4	REVERTED	QRK**T**VKCFNCGK	p7	A11
	MUTATED	-------**I**----------------		
5	REVERTED	NANPDCK**T**ILKALG	p24	A8, B7, B8, B35, B39, B51
	MUTATED	-------------**A**----------		
6	REVERTED	ELKSLYNT**V**AVLYF	p17	A2, A30, A68, B08, B57, B58, B63, Cw14
	MUTATED	----------------**I**---------		
7	REVERTED	DIAGTTS**T**LQEQIAW	p24	B7, B2705, B57, B58, B63
	MUTATED	-------------**N**-------------		
8	REVERTED	RLRPGGKK**K**YKL	p17	A3, A11, A30, A31, A68, B0702, B8, B27, B42, B51, B62
	MUTATED	----------------**R**-----		
9	REVERTED	GKK**K**YKLKHIVWAS	p17	A23, A24, A26, B8
	A) MUTATED	------**R**--------------------		
	B) MUTATED	------**R**--**R**--------------		

## Discussion

Transmission of HIV-1 virus is typically contingent on a single founder virus which generally depend on viral fitness [14–16]. CTL pressure selects for virus variants of variable fitness and these variants containing escape mutants can be readily transmitted [5, 17, 18]. Additionally, in perinatal transmission studies it has been possible to assess various aspects of CTL escape mutant targeting the outcome upon transmission from a known source [5, 19–21]. It has also been established that horizontal transmission to a new host that shares some of the same HLA types as the source results in impaired ability to recognize common CTL escape mutants [22]. However, reduced or abolished CTL pressure in a new host with a mismatched HLA background from the source may lead to reversion of previously selected CTL escape mutants [17, 23–25]. Some of these reversions represent the selection of consensus sequences that outcompete virus containing CTL escape mutations that result in substantially lower viral fitness. However, relatively little is known about the frequency with which T cell responses to the mutated CTL escape mutants following transmission between HLA mismatched adult individuals occur and where reversions may be due to new T cell pressures to virus containing the mutated sequences.

We have shown here that vigorous T cell responses are frequently directed against mutated epitopes, which subsequently were followed by reversions that abrogated or diminished T cell recognition in half of the individuals. These data confirm that one T cell escape mutation in an individual may often be immunogenic in another individual. We found that 9 of 52 reversions in CTL escape mutations were associated with immune responses to the mutation in the HIV-1 recipients, suggesting that about 17% of reversions of transmitted T cell escape mutations to consensus sequence may be driven by immune pressures directed at the escape mutation.

Immune pressure has been found to account for up to 40% of codon variations [26]. Several studies also show that polymorphism occurs in predictable patterns based on HLA-associated escape mutations. Furthermore, an extensive study identified and mapped amino acids that were found to be enriched or depleted in the presence or absence of specific HLA alleles [27]. However, T cell responses induced by these epitopes enriched and depleted of amino acids have not been extensively investigated. In the current study, we established that six of nine peptides containing *gag* epitope mutations in which we observed reversion to consensus sequences following transmission induced a T cell response in the new host, and that four of these six reversions resulted in diminished T cell recognition and IFN-γ production. This suggests that these reversions to consensus sequence likely represent T cell escape mutations in the new host. Responses to peptides containing the early targeted B7 epitope GPGHKARVL (GL9) (Peptide 1), were observed in two recipients. In both individuals the mutated sequence was recognized rapidly following transmission but decreased within a year of infection. Reversion did not result in diminished T cell recognition in these cases, suggesting that other factors, such as differences in viral fitness and compensatory mutations may account for selection of the reverted sequence.

One of the most commonly noted escape mutations, the T to N mutation at position 3 of the HLA B57 TW10 epitope (Peptide 7) was observed in 5 transmission pairs. This mutation has been shown to quickly revert in absence of HLA B57 or B58, presumably due to its high viral fitness cost [24, 25, 28]. However, responses to the TW10 escape variant have also previously been observed in HLA B57/5801 children following perinatal infection suggesting that immune escape in this epitope may result in variant-specific immune responses [19]. Similarly we demonstrated that there was a T cell response to the N-mutated version of an epitope within this region in HLAB57/5801 negative recipients, suggesting that in some cases a T cell response targeting a sequence in close proximity of the TW10 epitope may contribute to the high reversion rate.

The HLA A2-restricted immunodominant epitope SLYNTVATL (SL9) typically occurs late during infection and escape variants are not considered to affect T cell recognition or change viral fitness [5, 29–32]. Although SL9 has been shown to be targeted predominantly during chronic infection, we found that peptide 6, which contains SL9 variant V6I/T8V, induced a significant T cell response soon after transmission. Previous reports showed that presence of a 79F mutation in the upstream HLA A1-restricted CTL epitope GY9 reduce SL9 responses [31]. In this cohort 79F mutation was present in two transmission pairs where the sources were both HLA A1. Neither of the recipients in these pairs responded to the SL9 variant V6I/T8V or the reverted sequence, although only one of the recipients was HLA A2. The wild type peptide with a reversion at position 6 was not initially recognized, but during follow-up it subsequently induced IFN-γ production suggesting that a potential sequential broadening of CTL responses occurred which in early HIV-1 infection have previously been associated with viral escape [31]. Conversely, no responses to either the mutated or reverted peptide were detected in the source, even though this individual was HLA-A2. This indicated that T cell responses to this epitope may have waned, which may also explain the lack of responses in any of the sources to reverted peptides.

Responses to the sequence region containing the immunodominant HLA A3 epitope RLRPGGKKK (RK9, peptide 8) showed that despite reversion of R to K at position 9, T cell recognition was not abrogated. This supports previous observations where RK9 is an epitope that remains immunodominant over time. Similarly, the response to peptide 9, which contains the A24 epitope KYKLKHIVW (KW9), was only partially decreased following reversion. Only in the presence of an additional mutation with the reversion, as found in peptide sequence 9b, was T cell recognition significantly diminished.

In conclusion, this study highlights the intricate dynamics of HIV-1 polymorphisms in the presence of immune pressure, and emphasizes that immune pressure not only directly influences the occurrence of escape mutations, but may play a significant role in the reversion, and thereby restriction in polymorphism.
